# Hospital Chapels and Hospital Sunday

**Published:** 1917-06-23

**Authors:** 


### HOSPITAL CHAPELS AND HOSPITAL SUNDAY.

In some of the older endowed hospitals, and a few others, the chapel has occupied a prominent position in the plan of each institution, though the services fifty years ago and since have left something, and even occasionally much, to be desired. We rejoice to know that to-day most hospitals worthy of the name have a chapel which has come to occupy a helpful place in the daily life of its workers. Indeed, it has become the centre where the most earnest-minded and keen amongst them can find solace and refreshment, and where all unite on occasion to offer prayer and praise and to show by their presence their appreciation of the services and life of a dear colleague, or of respected and invaluable members of the staff. The Great War has added appreciably to the usefulness and welcome which the inmates of a modern hospital gratefully extend to the chapel, its services, and the mission of its clergy and ministers. At least a minority of hospital superintendents and matrons, from the earliest days, have recognised the aid which prayers in the chapel every morning contribute to the maintenance of a high standard amongst the workers, to the feeling of unity and discipline amongst them, and to the recognition of the sacred character of their work and its inspiration and helpfulness to all who take part in it.

So the hospital chapel has become a power for good. Indeed, where the chaplain has been a real live man, full of human feeling, with a generous and kindly disposition, he has ever been a welcome colleague to all responsible hospital workers. Looking back over decades of years of work in and for the hospitals, as the writer is able to do, he can gratefully testify to the influence, inspiration, and encouragement derived continuously by those, who form the habit of regular attendance at the services in the hospital chapel. Most hospital workers thus come to appreciate how essential it is that God's House, under the care of a good and capable man of God, should be, in fact, an active centre of vitalising influence and unity in every well-conducted hospital, whatever its field of work, irrespective of its size and relative position.

In this the Hospital Sunday Number of THE HOSPITAL, in the fourth year of the Great War, it seems well that the foregoing points in regard to the hospital chapel should be insisted upon, because the whole nation and Empire, through the war, have gradually awakened to a completer realisation of the spiritual nature of men and women, its needs and sustenance, than the oldest of us had experienced before in a lifetime. It is inconceivable, we are glad to believe, that to-day there can be a hospital chapel in the land, where the services are conducted in so perfunctory a manner as to make it necessary for the staff to seek spiritual consolation and comfort outside the hospital. This was once the case for the reasons we have already given. We hope that this position will be made by the preachers on Hospital Sunday, and that they will make a point of insisting that the modern hospital of every type, where our warriors have been and are being received, is in its daily and hourly work a living centre of testimony and recognition of Christ's teaching and Kingdom—a place where not only the bodies but the souls of men and women secure treatment and refreshment, which minister to their recovery here and their blessedness hereafter.

